# An automated process for bulk downloading optical coherence tomography scans

**DOI:** 10.1007/s00417-024-06420-1

**Published:** 2024-02-28

**Authors:** Yaacov Cnaany, Rivkah Lender, Itay Chowers, Liran Tiosano, Yahel Shwartz, Jaime Levy

**Affiliations:** Department of Ophthalmology, Faculty of Medicine, Hadassah University Medical Center, The Hebrew University of Jerusalem, 91120 Jerusalem, Israel

**Keywords:** OCT, Automated data extraction, Macro, VBA

## Abstract

**Objective:**

To develop an automated method for efficiently downloading a large number of optical coherence tomography (OCT) scans obtained using the Heidelberg Spectralis (Heidelberg Engineering, Heidelberg, Germany) platform.

**Methods:**

The electronic medical records and OCT scans were extracted for all patients with age-related macular degeneration treated at the Hadassah University Hospital Retina Clinic between 2010 and 2021. A macro was created using Visual Basic for Applications (VBA) and Microsoft Excel to automate the export process and anonymize the OCT scans in accordance with hospital policy. OCT scans were extracted as proprietary Heidelberg E2E files.

**Results:**

The VBA macro was used to export a total of 94,789 E2E files from 2807 patient records, with an average processing time of 4.32 min per volume scan (SD: 3.57 min). The entire export process took a total of approximately 202 h to complete over a period of 24 days. In a smaller sample, using the macro to download the scans was significantly faster than manually downloading the scans, averaging 3.88 vs. 11.08 min/file, respectively (*t* = 8.59, *p* < 0.001). Finally, we found that exporting the files during both off-clinic and working hours resulted in significantly faster processing times compared to exporting the files solely during working hours (*t* = 5.77, *p* < 0.001).

**Conclusions:**

This study demonstrates the feasibility of using VBA and Excel to automate the process for bulk downloading data from a specific medical imaging platform. The specific steps and techniques will likely vary depending on the software used and hospital constraints and should be determined for each application.
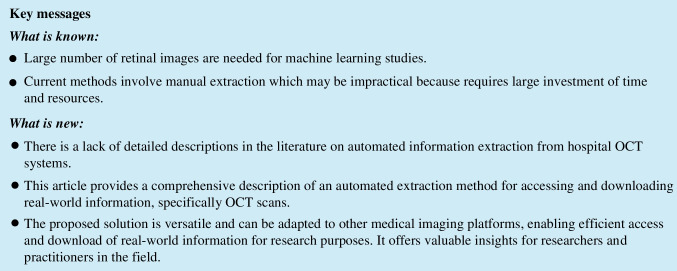

## Introduction

Optical coherence tomography (OCT) is a non-invasive imaging technique that uses low coherence light to provide high-resolution images of retinal structures. The past several decades have seen significant growth in the capabilities of new and existing imaging modalities, and OCT is a clear example of this progress, as OCT can now provide images at a resolution of approximately 10 µm/pixel in lateral scan mode [1, 2]. Due to its high resolution, this imaging modality has several applications in the field of ophthalmology, particularly the ability to obtain high-resolution images of retinal structures for diagnostic purposes. Indeed, OCT has revolutionized the clinical management of many retinal diseases, including age-related macular degeneration (AMD) [3–5], diabetic retinopathy [6–8], and retinal vein occlusion [9, 10]. Over the past two decades, OCT has become the most commonly used imaging technique in ophthalmology; in 2017, approximately 6.74 million scans were performed in the USA on Medicare recipients alone [3, 11, 12]. In chronic retinal pathologies such as AMD, individual patients can receive follow-up care as frequently as every few weeks over the course of years [13, 14], resulting in vast amounts of imaging data. This provides the ideal opportunity to develop artificial intelligence (AI)‒based tools to support clinical decision-making, with the majority of these tools based on deep learning and/or computer vision (in which a computer derives information from images, videos, or other inputs) [12, 15]. Many researchers, however, consider it a major obstacle when it comes to acquiring large imaging datasets [12].

The majority of medical imaging platforms allow users to download single images via various software programs. However, when it comes to the relatively large number of images required for use in the field of data science, this process is impractical and requires a large investment of time and resources that can otherwise be invested in performing the research itself. Thus, although automated methods are needed in order to facilitate the large-scale bulk download of medical images, only a few imaging modalities currently provide a “bulk export” feature [16].

Studies have shown that combining deep learning with OCT scans can help detect retinal disease and can help determine the urgency of ordering a referral for patients with potentially sight-threatening ocular conditions [17–19]. Here, we describe a new method to address the difficulties associated with bulk downloading images for a defined study group.

The Retinal Clinic in the Hadassah Ophthalmology Department is currently conducting a study involving patients with AMD using a combination of AI and big data techniques in order to improve both our understanding of AMD and our medical diagnostic capabilities. The study group was extracted from the hospital electronic medical records (EMRs) database based on ICD-9 (International Statistical Classification of Diseases and Related Health Problems, version 9) codes. As part of this study, a technical team at the hospital retrieves the patients’ clinical data from the EMR systems using business intelligence techniques; these data include the patients’ medical follow-up information and clinical information. However, the team encountered a challenge when attempting to retrieve all of the high-resolution OCT scans for the patients in the study, as the OCT device used (Heidelberg Spectralis OCT version 1.10.4.0, Heidelberg Engineering, Heidelberg, Germany) does not provide the ability to batch export data. To overcome this issue, we attempted to develop an automated process that can export these OCT scans quickly and efficiently. Using this process, all patient identifiers were encrypted in accordance with hospital policy, the Helsinki Committee, and the Israeli Ministry of Health (HMO-19–0382). Moreover, the patient files had to be anonymized as well as downloaded, which required an additional set of automated steps in the process.

## Methods

Hospital’s IT security constraints often prevent the installation and use of common external programming languages such as Python and R ​​in their computer systems. To overcome this limitation, one study described a solution using JavaScript [20]. To develop a more user-friendly and more readily accessible solution, we wrote a macro for Microsoft Excel using Microsoft Office’s programming language Visual Basic for Applications (VBA), which is part of many Microsoft Office applications and is already integrated in most modern hospitals. The VBA script code can be accessed via a GitHub repository (https://github.com/kobicna/HEYEX1-OCT-E2E-Downloader/).

We found that the VBA user interface (UI) automation techniques used in the macro allowed for a more efficient and streamlined process for exporting OCT files from the Heidelberg Spectralis software. Microsoft’s UI automation tool allows users to access, identify, and control the UI elements of other applications via an application programming interface (API). To ensure optimal performance, the macro’s script receives an Excel file containing a list of patient identification numbers and their corresponding codes for anonymization purposes while the Heyex OCT imaging software is opened in full-screen mode and the screen resolution is set to 1920 X 1080. By recognizing pixel color values at various x,y coordinates, combined with predefined locations on the screen and a search for titles using user interface (UI) automation, the script was able to identify the various menus presented by the software. Thus, by automating the interactions with the UI and by controlling mouse cursor movement and keystrokes, the script was able to rapidly navigate through the various menus and initiate the export process. In addition to saving both time and user effort, this process also reduced the risk of errors that can occur when users manually export the data. In addition, the script also ensured that all patient information was properly anonymized in accordance with the study protocol, an important process in which any potentially identifying information is removed or obscured from the images before being used in research or other applications, thereby protecting patient privacy and ensuring compliance with both ethical and legal guidelines. In the context of research, image anonymization is necessary in order to ensure that the data being used lack any identifying information and cannot be traced back to individual patients, and is often required by institutional review boards (IRBs) and other regulatory bodies as a condition for approving the research protocol.

To ensure that all exported files were properly anonymized and protected patient privacy, the script used the anonymization codes provided in the input Excel file, encrypting key identifiers such as the patient’s name, ID number, and date of birth. The output Excel file generated by the script provided a detailed record of the export process for each subject, including information such as the anonymized patient ID, file location, number of files, total file size, and export start and finish times. This information was useful for tracking the export process and identifying any issues that may have occurred. If no images were available for a particular subject, the Excel file included only the anonymized ID, indicating that no files were exported for that subject.

It is worth noting that using the script to download OCT scans during the clinic’s regular operating hours led to issues such as bandwidth overload and delays in patient care, which affected the clinic’s ability to provide timely and efficient care to patients. To address this important issue, we modified the system so that it would operate primarily outside of the clinic’s normal operating hours. This change reduced the burden placed on the OCT system and reduced the potential impact on patient care.

**Fig. 1:**
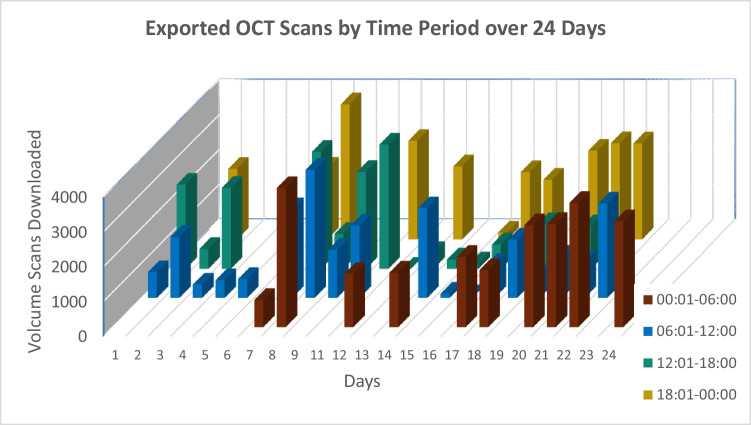
Summary of the number of files exported over 24 consecutive days using the automated download system, with each day divided into four 6-h time periods. Initially, using this the system in the mornings (from 06:01 to 12:00) reduced the bandwidth available for use in the clinic. Therefore, starting on day 12 the remaining files were downloaded primarily off-clinic hours (i.e., from 18:01 to 06:00 on weekdays and during the weekend)

We exported OCT data for 24 consecutive days. Figure 1 summarizes the number of files exported each day of the process, with each 24-h day divided into four equal time periods (00:01–06:00, 06:01–12:00, 12:01–18:00, and 18:01–24:00). Note that the daily number of files exported from 12:01 to 18:00 dropped considerably after day 11, as running the script during peak clinic hours slowed the clinic’s workflow; thus, starting on day 12 the majority of the remaining files were exported during off-clinic hours (i.e., between 18:01 and 06:00).

We also analyzed a small sample in order to compare the average download time per file between manual export and our automated method. To ensure a fair comparison, we performed a manual download based to the script’s workflow using a random sample of five patients with a total of 132 OCT volume scans and then calculated the average download time per scan.

### Statistical analysis

Processing time was treated as a continuous variable, and differences between manual and automated extraction were analyzed using a non-paired Student’s *t* test (IBM SPSS Statistics version 26).

## Results

Of the 4168 patients in the AMD study group, a total of 94,789 E2E files (totaling 4849.07 gigabytes, about 10% of the total Heidelberg database) were obtained for 2807 patients with AMD scanned using Heidelberg Spectralis OCT devices (Heidelberg Engineering Inc, Heidelberg, Germany). The volume scans consist of horizontal raster lines, and the data were saved in Heidelberg’s proprietary E2E file format. Collaborating with a Computer Science engineer from the Hebrew University of Jerusalem, we performed additional analysis and converted the files to TIF using C +  + library from an open-source GIT repository [21]; to the best of our knowledge, the version of the software that we used can output several scans for a single patient only if the files are saved as this file type. Using one computer, the automated export process took slightly under 202 h over 24 days, with the process performed primarily during weekends and off-clinic hours (Fig. 1). The average patient EMR contained 37 E2E files (i.e., volume scans) per eye, resulting in a mean download time of 4.54 min (SD: 3.57 min).

We found that the average download time was significantly shorter during off-clinic hours compared to during clinic hours, with an average scan download time of 3.97 ± 3.10 min vs. 4.75 ± 4.05 min, respectively (*t* = 5.77, *p* < 0.001). Thus, the scans were downloaded approximately 20% faster during off-clinic hours, indicating that downloading OCT scans during these hours resulted in a savings of approximately 48 s per scan. This difference in download time was likely due to the reduced use of the hospital’s computer system during off-clinic hours, allowing the system’s full bandwidth to be used for automated downloads.

Our subsample analysis revealed that our automated process was significantly faster than manually downloading the scans. Specifically, our automated method took an average of 3.88 min to download a patient’s scans compared to 11.08 min for manual downloads (*t* = 8.59, *p* < 0.001). Moreover, the average time to download a single scan file using the automated system was 8.830 ± 6.850 s compared to 25.18 ± 20.740 s for manual download, an average savings of 16.350 s per file. These results demonstrate that our automated script-assisted method for downloading OCT volumes scans is significantly more efficient than manually downloading the scans.

## Discussion

Here, we report the feasibility of using VBA and Excel to automate the process for bulk downloading images obtained using the Heidelberg Spectralis OCT platform, an imaging device commonly used in ophthalmology clinics [12]. Although a newer version of the Heidelberg software called Heyex2 does offer a bulk download feature [22], this version is not available in many clinics—including ours. An independently written script may provide more flexibility and control over specific parameters compared to commercial software. Moreover, similar tools have been developed for exporting radiology images; for example, Draelos et al. used an API originally developed for Duke University’s vendor-neutral archive to query and download 36,316 chest CT scans [23]. Finally, with respect to exporting OCT scans, only one study was reported in which a script was written in JavaScript and used to export 72,894 OCT scans [20].

Prior to the development of our automated download process, manually exporting approximately 100 patient files required two of our researchers working full-time for approximately 1 month; using our automated process, the files of more than 2000 patients can be downloaded in the same amount of time. Thus, manually downloading OCT data is a needless waste of resources and can even impede progress. In contrast, using automated processes such as the one described here can help accelerate research by utilizing the vast quantities of available data for developing more advanced and rational approaches to medicine.

In this study, our principal aim was to compare average OCT file download times between manual and script-assisted methods. Manual methods require the labor-intensive, time-consuming, and repetitive process of searching patient EMRs, identifying a suitable patient, and then manually downloading and encrypting the E2E files, a process described as tedious by our research team. On the other hand, our script-assisted automated method does not require human intervention and can be performed outside of the clinic’s peak hours, thereby significantly accelerating the download process.

Building on previous studies [24, 25], our results indicate that VBA and Excel can be used to automate the bulk image download process when applied to a specific medical imaging platform. This method has the potential to greatly accelerate the collection of large-scale data for research purposes, particularly in situations in which other programing languages such as Python and R are either not available or not practical. Nevertheless, the specific steps and techniques used here may differ depending on the specific medical imaging software used and the hospital’s constraints regarding information security. In addition, the use of VBA and Excel may not necessarily be the most efficient or effective method for downloading images in all situations, and further research is needed in order to compare the performance and limitations of other approaches.

The development of AI for supporting the clinical decision-making process using large imaging datasets [26] has the potential to revolutionize the way in which retinal diseases are managed. For example, deep learning algorithms have been shown to perform quite well in terms of detecting retinal diseases and determining the urgency of referring patients with potentially sight-threatening ocular conditions [17–19]. Developing and validating machine learning algorithms for medical image analysis necessitates the use of extensive and high-quality datasets. However, many research groups rely on known datasets or manually acquire small numbers of scans [26–28]. While open datasets are available, they may not accurately reflect the real-world data or allow for working with information gathered during routine clinical visits, including treatments. Therefore, the automated method presented in this study enables researchers to collect large, representative datasets of clinical data from their local clinic/hospital. This approach can be useful for developing AI-based tools for use in ophthalmic devices and other medical imaging devices that lack a bulk download feature. By accessing clinical data from the EMR system, researchers can combine visits, including all available information on the patients, and match real-world information to facilitate the development of accurate and robust algorithms for medical analysis.

**Fig. 2:**
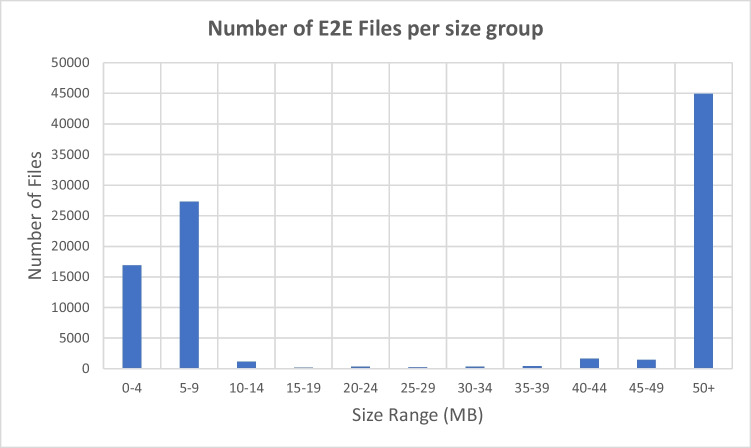
Distribution of the size of the exported files

Despite its advantages, the current script-based tools have several limitations that warrant discussion. First, only the entire patient record can be exported. Second is the inability to selectively export images or choose specific dates for the imaging data. Consequently, image reports, which contain personal patient data despite anonymization, were included in the exported files. To mitigate this issue, we filtered the patient files by size and retained only those larger than 5 MB, as our subsample analysis indicated that files smaller than 5 MB had a higher probability of containing patient-identifying information (Fig. 2). Third, depending on the available bandwidth continuous bulk download of files can slow the performance of open computer stations, which can cause problems if used during the clinic’s working hours. However, we found that running the script primarily during evenings and weekends—when the clinic is less active—significantly accelerated processing time and reduced interference during working hours. Fourth, this study pertains to the lack of evaluation of download accuracy between the automated and manual approaches. The script was designed to extract information expeditiously, but without consideration of potential discrepancies in accuracy. While manual downloads may be more precise, they are also more time-consuming. An advantage of automated approaches is that filtering of pertinent data can be executed programmatically after transfer to more advanced computational resources. However, it is important to emphasize that the script also ensures compliance with privacy policies by stripping personal information from the data.

In conclusion, we report that VBA and Excel can be used to automate the bulk download of images obtained using a specific medical imaging platform. In the future, this method can be adapted for use in other applications both in ophthalmology and in other medical fields. Further research is clearly needed in order to compare performance and limitations among various methods for bulk downloading images, and to weigh the potential benefits against the ethical and legal implications of using large datasets for clinical decision-making.

## Data Availability

The script can be found at https://github.com/kobicna/HEYEX1-OCT-E2E-Downloader/.
